# Novel Hybrid Scheduling Technique for Sensor Nodes with Mixed Criticality Tasks

**DOI:** 10.3390/s17071504

**Published:** 2017-06-26

**Authors:** Mihai-Victor Micea, Cristina-Sorina Stangaciu, Valentin Stangaciu, Daniel-Ioan Curiac

**Affiliations:** 1 Department of Computers and Information Technology, Politehnica University of Timisoara, V. Parvan No. 2, Timisoara 300223, Romania; mihai.micea@cs.upt.ro (M.-V.M.); cristina.stangaciu@cs.upt.ro (C.-S.S.); valentin.stangaciu@cs.upt.ro (V.S.); 2 Department of Automation and Applied Informatics, Politehnica University of Timisoara, V. Parvan No. 2, Timisoara 300223, Romania

**Keywords:** real-time scheduling, control system, hard real-time system, mixed-criticality system, hybrid scheduling, schedulability analysis

## Abstract

Sensor networks become increasingly a key technology for complex control applications. Their potential use in safety- and time-critical domains has raised the need for task scheduling mechanisms specially adapted to sensor node specific requirements, often materialized in predictable jitter-less execution of tasks characterized by different criticality levels. This paper offers an efficient scheduling solution, named Hybrid Hard Real-Time Scheduling (H^2^RTS), which combines a static, clock driven method with a dynamic, event driven scheduling technique, in order to provide high execution predictability, while keeping a high node Central Processing Unit (CPU) utilization factor. From the detailed, integrated schedulability analysis of the H^2^RTS, a set of sufficiency tests are introduced and demonstrated based on the processor demand and linear upper bound metrics. The performance and correct behavior of the proposed hybrid scheduling technique have been extensively evaluated and validated both on a simulator and on a sensor mote equipped with ARM7 microcontroller.

## 1. Introduction

Despite their exponential growth in many application areas, wired or wireless sensor networks have yet to make a breakthrough in safety- and time-critical domains where predictability, determinism and compliance with tight time constraints are seen as core features. A key reason for this is the lack of reliable and flexible scheduling algorithms to manage the plethora of tasks, with diverse criticality levels, raised at the sensor node level and on the network level as well. 

Task scheduling is considered a key mechanism to effectively ensure the imposed performances of time- and resource-constrained sensor nodes, but in the case of mixed criticality applications its role becomes even more crucial. Here, the scheduling policy is governed by task criticality class membership, so an accurate preliminary task classification is therefore needed. While task scheduling is usually described as a Nondeterministic Polynomial time-complete (NP-complete) optimization problem [1], finding its optimal solution is generally not operationally feasible. In this context, a series of algorithms attempting to provide near-optimal solutions can be suitable candidates.

Accordingly, the scheduling approaches designed for mixed criticality systems (MCSs) can be categorized as either static, when the scheduling decisions are done offline before the system starts running, or dynamic, where the scheduling decisions are made during runtime at different time moments called scheduling points. While the static approaches are highly predictable and reliable, but very inflexible, the dynamic algorithms are flexible, but subject to a certain degree of unpredictability. 

In our view, a carefully designed ensemble made of one static non-preemptive, table driven scheduling algorithm called Fixed Execution Non-preemptive (FENP) algorithm [2] and one dynamic, derived from classic Earliest-Deadline-First (EDF) algorithm [3], can ensure the efficiency of the task scheduling mechanism, in terms of flexibility and maximum CPU utilization factor (compared to an approach based only on clock-driven scheduling mechanism), while preserving the determinism and stability of the static schedulers. Moreover, we have designed a new and effective theoretical separation method between the proposed criticality levels, each level having its own execution environment, isolated from the others. The proposed approach offers certain advantages in the case of sensor nodes (e.g., timely and unperturbed execution of periodic tasks by simply placing them in the highest criticality class), which were validated through intensive studies that had been carried out both on an experimental platform (a sensor mote equipped with an ARM7 processor) and by computer simulation.

The rest of the paper is organized as follows. Section 2 surveys the related work and highlights the salient features of the proposed approach. In Section 3, we formalize the scheduling problem by presenting the system model, along with the notations and assumptions used throughout the paper. Section 4 describes in detail our hybrid scheduling algorithm, named Hybrid Hard Real-Time Scheduler (H^2^RTS), designed as a combination between FENP, which is a variant of non-preemptive, static, highly predictable, table driven scheduling algorithm, and the MEDF (Modified Earliest Deadline First) dynamic scheduler. A thorough theoretical integrated schedulability analysis of the H^2^RTS scheduler is performed in Section 5, while the performances of the proposed technique are evaluated in Section 6 using the results of extensive simulations (over 22,000 hard real-time task sets) and the measurements performed on a real sensor mote with hard real-time operating specifications. Finally, Section 7 concludes this paper.

## 2. Related Work

The utilization of wired or wireless sensor networks in hard time-constrained applications is limited by software and hardware capabilities of sensor nodes. While the hardware progress is rapidly accelerating, the development of new in-network information processing techniques adapted to such time-critical applications remains a significant challenge for both researchers and practitioners. Typical sensor networks are endowed by operating systems that provide no or limited support for real-time applications [4,5]: TinyOS employs a simple non-preemptive First-In-First-Out (FIFO) scheduling algorithm [6]; Contiki is an event-driven operating systems using priority based interrupts [7]; LiteOS uses a priority-based process scheduling algorithm [8]; and the MultimodAl system for NeTworks of In-situ wireless Sensors (MANTIS) provides very limited support for real-time applications using a preemptive priority-based scheduling with priority classes [9]. However, in the scientific literature, two notable exceptions have been reported: (a) Nano-RK [10], which is a real-time operating system that implements a priority driven fully preemptive scheduling algorithm; and (b) MIROS, which employs a multithreaded scheduling model based on the RMS (Rate Monotonic Scheduling) [5]. The central drawback of these real-time operating systems, which limits their application in practice, is that the tasks priorities are set only statically.

Our current research focuses on the development and analysis of a hybrid scheduling mechanism to efficiently tackle the task prioritization process in the context of mixed-criticality applications specially tailored for sensor nodes. The proposed mechanism combines two scheduling policies: one which is static, structured cyclic and non-preemptive, and the other one which is dynamic and priority-based. The main goals of this scheduling technique are to bring the advantages of the dynamic approach (flexibility and higher processor utilization ratio), while preserving at the same time the high predictability provided by the static scheduling policy.

To provide an efficient task scheduling algorithm for sensor nodes operating under hard time constraints we appeal to some of the lessons already learned in scheduling the tasks on embedded systems. In this respect, real-time task scheduling was envisioned as a complex optimization problem. For more than two decades, researchers have mainly focused on general and heavily constrained optimization problems including the representative model of hard real-time systems where the scheduling process is almost pushed to the limit. Recent years have proven an increasing trend to simplify the general scheduling optimization problem without adversely affecting the scheduling efficiency and flexibility by introducing reasonable implementation-specific assumptions. A relevant example in this regard is represented by mixed criticality systems where the tasks are confined into a finite number of categories described by criticality level values.

From this perspective, general scheduling techniques represent a valuable starting point to design efficient MCSs task scheduling for sensor nodes. While mixed criticality systems have particular features that must be carefully used to define optimal schedulability policies, a possible solution is to particularize general hard real-time scheduling algorithms for coping with a finite number of task criticality levels. From the plethora of such algorithms that may be considered as suitable candidates, until now two have gained particular attention from the research community: earliest deadline first (EDF) and heterogeneous earliest finish time (HEFT). Among these two algorithms, EDF is by far the most popular, both in classic real-time systems and in MCSs. Some of the outstanding EDF based scheduling mechanisms developed in the context of MCSs are: (i) EDF with Virtual Deadlines (EDF-VD), a scheduling algorithm initially proposed by Baruah et al. [11] for uniprocessors and later extended to multiprocessor systems by Li and Baruah [12] with the help of another EDF based scheduling algorithm that is used at the global level, called fixed priority EDF (fpEDF) [13]; (ii) Mixed Critical EDF (MCEDF) [14], an algorithm which uses EDF to improve the performance of a popular mixed criticality scheduling algorithm called Own Criticality-Based Priorities (OCBP) [12], by determining different scheduling priorities for different criticality levels for the same tasks; (iii) Task Grouping EDF (TG-PEDF) [15], an algorithm which targets multiprocessor systems using a mixed criticality uniprocessor scheduling strategy based on task grouping, which then schedules the groups with EDF while the tasks within a group are prioritized using a server-based scheduling algorithm; and (iv) MxC-RUN [16], another partitioned hierarchical scheduling for multiprocessor MCS which uses EDF fixed rate scheduling servers.

To cope with ever-increasing task heterogeneity, a series of relevant hybrid scheduling approaches have employed carefully chosen ensembles of scheduling algorithms. From this perspective, three interesting methods attracted our interest: (a) an effective method to combine the offline scheduling techniques with the online ones which is based on the conversion of the offline schedules into task attributes for fixed priority scheduling [17]; (b) an approach for hybrid scheduling proposed in [18], where the tasks are divided in sets according to some common features and later scheduled within their own set (each set can have its own scheduling algorithm); and (c) a hybrid preemptive scheme known as Earliest Deadline Zero Laxity (EDZL) [19] which proposes the use of the global EDF algorithm until one of the tasks reaches zero slack (or zero laxity). In this circumstance, the priority of the mentioned task is immediately raised to the highest level.

While some preliminary research results, obtained in the first stage of this project, were offered in [20], the present paper presents the fully functional H^2^RTS algorithm, an efficient combination between FENP and MEDF.

## 3. System Model, Notations and Assumptions

When speaking about mixed criticality systems, a series of less or more general system models have been reported during the last decade. The first one was proposed in [21], describing the MCS task model as having different computation time for every design assurance (i.e., criticality) level and was later adapted to capture diverse facets of the mixed-criticality scheduling process [22,23]. The model that we adopt in this paper derives from the classical model of hard-real time systems, with tasks being classified into scheduling classes based on their criticality.

Thus, we consider a model ***S*** of hard real-time system featuring mono-processor execution support for a statically defined set of *N* tasks, ***S*** = {*τ*_1_, *τ*_2_, ..., *τ_N_*}. The timing behavior of each task *τ_i_* is specified by the following main parameters:

- *T_i_*: the period, if *τ_i_* is periodic, or the minimum inter-arrival time of its jobs, if *τ_i_* is a sporadic task;
- *C_i_*: the computational cost of *τ_i_*, defined as the worst-case execution time (WCET) among all jobs issued by *τ_i_*;
- *D_i_*: the relative deadline of *τ_i_*, defined as the maximum allowable response time of any job generated by this task;
- *U_i_*: the worst-case processor utilization factor of *τ_i_*, defined as the ratio *C_i_*/*T_i_*; the system (total) utilization factor is denoted as *U* = ∑*_i_U_i_*; and
- In addition to these parameters we included another parameter to describe the criticality level:
- *L_i_:* the criticality level*.*

We considered three criticality levels, which are statically assigned in an offline mode, “*extreme*”, “*high*” and “*low*”, and we assume that once assigned a criticality level for a task, this cannot be changed during runtime. 

We assume all the tasks in ***S*** have deadlines not larger than their respective periods, i.e., *D_i_* ≤ *T_i_*. Although we assume no restrictions regarding the release times of the tasks, in this work we approach the case when all the tasks have the same release time instant—the so-called “critical instant for the task system” [24].

To simplify the discussion, all tasks are assumed independent to each other. This would not restrict the generality of the treatment as control and data dependencies can be supported through various state of the art techniques, e.g., guarded buffers, static structures for task I/O parameters [25] or other types of protected objects. In the same time, task dependencies and potential resource access contentions will also be solved by the particularities added to this task model and by the scheduling strategy of the proposed method, as presented in the following sections.

Finally, we consider scheduling techniques without inserted idle time, i.e., the processor will not be allowed to enter idle state as long as there are active jobs which have not completed execution. Priority-based scheduling will be used; therefore a priority is assigned to each task, either statically or dynamically, and the processor will be allocated to the active job with the highest current priority.

## 4. H^2^RTS: The Hybrid Hard Real-Time Scheduling Algorithm

A key set of goals providing the basis for developing the H^2^RTS (Hybrid Hard Real-Time Scheduling) technique, are presented in this work:

- Providing the system predictability and timeliness even under worst-case operating conditions;
- Maximizing the efficiency of highly predictable scheduling techniques, regarding mainly the processor utilization factor;
- Reducing the schedulability analysis effort (time and complexity); and
- Keeping the system overhead introduced by the online task scheduling and execution mechanisms at a low value.

These principles derive from the shortcomings of many approaches in the state of the art in the field of critical and hard real-time applications, as pointed out in the first two sections of the paper.

As a hybrid technique, H^2^RTS combines the predictability of a variant of non-preemptive, cyclic (table-driven) scheduling algorithm, called Fixed Execution Non-Preemptive (FENP), with the efficiency of the Earliest Deadline First scheduler (actually, a modified EDF version (MEDF), which will be further discussed in this section). Therefore, the task system ***S*** will statically be split into two corresponding subsets, according to their criticality level:

***S*** = {***S**^FENP^*, ***S**^MEDF^*} = {*μ*_1_...*μ*_m_, *ε*_1_...*ε*_n_}. *m* + *n* = *N* (1)

Each task from the ***S****^FENP^* set has its criticality level equal to “extreme” and each task from the ***S****^MEDF^* set has its criticality level equal to “high”. These two types of tasks are often encountered in control applications and they are similar with the ones proposed in [26] for a mixed-criticality automotive software: the “safety-critical control applications with stability and performance constraints” can correspond to ***S****^FENP^* subset of tasks and the “time-critical applications with only deadline constraints” can correspond to ***S****^MEDF^* subset of tasks.

### 4.1. FENP Scheduling Algorithm

The Fixed Execution Non-Preemptive (FENP) algorithm [2] has been designed to provide maximum predictability for the execution in a high-priority, non-preemptive context, of periodic tasks with the highest criticality. It has been implemented on the HARETICK kernel [27] and used in various real-time signal acquisition, processing and data communication applications which require perfectly synchronized interactions with signals [28,29].

The FENP name derives from the cyclic execution paradigm, i.e., all jobs of a task are executed at a fixed time instant within their respective periods. Thus, the execution of any two consecutive jobs of a task *μ_i_* in ***S****^FENP^* is separated by time intervals of the same length, equal to the task period:

*s_i_*_,*k*+1_ = *s_i_*_,*k*_ + *T_i_*. 1 ≤ *i* ≤ *m* (2)

Because of Equation (2), the start time of any execution instance (job) of *μ_i_* can be statically determined in a direct manner:

*s_i_*_,*k*+1_ = *s_i_*_,1_ + *kT_i_* = *φ_i_* + *kT_i_* (3)

where *φ_i_* is the start time of the first job of *μ_i_* (also called the “phase” of *μ_i_*). Equations (2) and (3) formally describe the scheduling principle of this algorithm: the start time of the next instance (*k* + 1) of a FENP task *μ_i_* is computed by adding the task period *T_i_* to the start time of the current instance, *s_i_*_,*k*_.

Another particular feature introduced by the FENP method to the task model presented in the previous section is that the relative deadlines are equal to the corresponding periods:

*D_i_* = T_i_, ∀μ_i_ ∈ ***S**^FENP^* (4)

As a result, the timing behavior of a task *μ_i_* in the ***S****^FENP^* subset can be fully determined by the tuple.

*μ_i_* ≡ (*φ_i_*, *C_i_*, *T_i_*). 1 ≤ *i* ≤ *m* (5)

Without affecting the generality of the approach, we consider that the tasks in the ***S****^FENP^* subset are arranged in non-decreasing order of their periods.

*T_i_* ≤ *T_i_*_+1_. 1 ≤ *i* ≤ *m* − 1 (6)

To summarize, the tasks in the ***S****^FENP^* subset are executed in a perfectly periodic manner (considering the start times and the relative deadlines of their respective jobs), with the highest precedence in a non-preemptive highly critical context. 

The schedulability analysis is based on an efficient offline test, which also determines the relative start time (phase *φ_i_*) of each task *μ_i_* in ***S****^FENP^*, if the subset has been found feasible [2]. Even though the FENP has been developed for (perfectly) periodic tasks, the algorithm can also be used for scheduling sporadic tasks using the so-called “Ghost ModXs” or “ghost jobs”, which are defined and described in [27].

### 4.2. MEDF Scheduling Algorithm

The FENP scheduling algorithm has two major drawbacks, though. First, there is its lack of flexibility, as with the case of all the fixed-priority algorithms which statically assign the priority of tasks at the offline analysis phase, based on their respective time parameters. The “ghost job” and “execution counter” mechanisms [27] provide a partial solution by solving the case in which a period of a task needs to be modified at runtime by multiples of the initial period. The second shortcoming of the FENP algorithm is its lack of efficiency regarding the maximum total processor utilization factor, which is typically below 70%. The solution proposed here is to combine the FENP technique with a more efficient scheduling algorithm, which is also able to provide hard timing guarantees. Thus, only those application tasks needed to operate in a perfectly synchronous mode will be scheduled with the FENP technique. We focused on the Earliest Deadline First (EDF) scheduling algorithm, which is optimal, both in preemptive and in certain non-preemptive contexts, with respect to task schedulability, as well as to the processor utilization efficiency [30,31,32,33,34].

To increase the system predictability, a modified version of the EDF algorithm has been introduced (denoted as MEDF). The major difference from the classic technique is the restriction that the tasks in the ***S****^MEDF^* subset cannot preempt each other; they can be preempted only by tasks from higher criticality contexts (i.e., the FENP context, in our case). This approach eliminates unpredictability issues related to nested context saving and switching, synchronization and inter-task communication, as well as to task blocking and priority inversion phenomena generated by potential access contentions to shared resources.

MEDF dynamically schedules the tasks in ***S****^MEDF^* by selecting each time the job which is ready (e.g., has not been executed in its current period) and has the earliest deadline with respect to the current scheduling time. Due to the specific preemption restrictions, the scheduling decisions need to occur only at the completion of each MEDF job, to establish the next job to be executed, along with its corresponding start time. Similar to Equations (2), (3), (5) and (6), the MEDF scheduling algorithm can be formally described by the following system:

(7) $$\{\begin{array}{l} S^{MEDF}=\left\{\;\varepsilon_{1},\;\ldots ,\;\varepsilon_{j},\;\varepsilon_{j+1},\;\cdots ,\;\varepsilon_{n}\right\}\\ \varepsilon_{j}\equiv \left(C_{j},\;T_{j},\;D_{j}\right)\;.\;D_{j}\leq T_{j},\;j=1..\;n\\ D_{j}\leq D_{j+1}\\ \begin{array}{l} s_{j,\;k_{j}+1}=\max\left(t,\;k_{j}T_{j}\right)\;,\;\mathrm{where}\text{:}\\ \;j\;|\;\forall p\neq j,\;\left(k_{j}T_{j}+D_{j}-t\right)\leq \left(k_{p}T_{p}+D_{p}-t\right)\\ \;\mathrm{and}\;k_{j},\;k_{p}\in N \end{array} \end{array}$$

where the scheduling mechanism is stated in the last relation. It is based on the current decision time, *t*, when the next task is selected for scheduling. The next task will have the index *j*, if

(a) it has not been already executed within its period (the last executed instance is *k_j_*); and
(b) its absolute deadline is the closest to *t* among all the other MEDF tasks which are also ready. After selecting the task to be scheduled next (*ε_j_*), its start time will either be the current moment (*t*), or the start of its next period (*k_j_T_j_*), if the current period has not elapsed yet.

### 4.3. H^2^RTS Hybrid Scheduling Method

H^2^RTS assumes a static partitioning of the hard real-time tasks into two subsets, according to (1):

(a) The first subset, ***S**^FENP^* ≡ {*μ*_1_, *μ*_2_, ..., *μ_m_*}, contains the tasks with perfect synchronous operating requirements, which are scheduled with the FENP algorithm, and executed in the highest priority, non-preemptive context, a context which can be considered the highest criticality level of the system (marked as “extreme” in the task model).
(b) The second subset, ***S**^MEDF^* ≡ {*ε*_1_, *ε*_2_, ..., *ε_n_*}, also consists of tasks with hard timing specifications, but which do not require perfect periodic operation. They are dynamically scheduled with the MEDF algorithm and are executed in a semi-preemptive context, i.e., any active *ε_j_* task can be preempted only by tasks in the FENP context, which represents a higher criticality level. This context is also a high criticality context (marked as “high” in the task model), but having a smaller priority compared to the FENP context.
(c) Additionally, we consider a third execution context (“background context—BGND”), of the lowest priority and criticality level (marked as “*low*” in the task model), which accommodates tasks with soft or without any timing specifications. These are scheduled using traditional multitasking/multi-threading techniques with or without priorities, such as round-robin or multilevel queue algorithms. Further details regarding this context are out of the scope of this paper and, hence, our discussion resumes to the task system in Equation (1).

It is worth noticing that, usually, perfectly periodic hard real-time tasks are a minority among the other application tasks (*m* << *n*) and, thus, the efficiency of the FENP scheduling will have a relatively low impact on the overall system efficiency.

**Figure 1:**
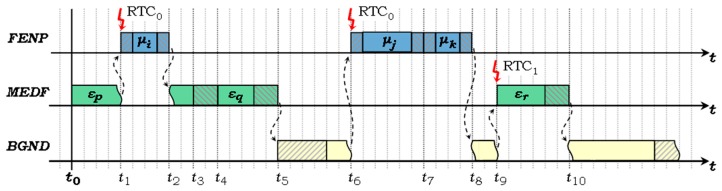
Execution contexts and system operation under the H^2^RTS method.

Figure 1 exemplifies the system operation within its three execution contexts. Suppose at instance *t*_0_ the MEDF task *ε_p_* starts its execution. At *t*_1_, the task *μ_i_*, from the higher priority FENP context, is scheduled to start, based on the interruption issued by the real-time clock RTC_0_. Therefore, *μ_i_* interrupts the execution of *ε_p_*, saves its context, and takes over the system processor. Upon completion of *μ_i_* at instance *t*_2_, the start time of the next scheduled FENP task is programmed into RTC_0_, then the MEDF context is restored and *ε_p_* resumes execution.

The context switching and the real-time clock programming operations previously mentioned are performed by the FENP dispatch mechanism, implemented as two components, a prefix and a suffix, which frame the executions of each FENP task. The FENP dispatch components are shown in Figure 1 with shaded rectangles around *μ_i_*, *μ_j_* and *μ_k_*.

Task *ε_p_* finishes at *t*_3_ and, consequently, the next MEDF task to be scheduled, is decided at the same time instance. The MEDF scheduler is depicted in Figure 1 with shaded rectangle. In this example, *ε_q_* is ready and has the earliest deadline; therefore, it will be launched at *t*_4_. Upon completion, the MEDF scheduler decides the next task to be executed, *ε_r_*, will be ready at instance *t*_9_. It programs the second system timer, RTC_1_, to issue an interrupt at *t*_9_. During this idle interval, the processor is released to the lower priority context, the BGND. Here, the corresponding scheduler (shaded in Figure 1) and tasks are launched. At *t*_6_, the BGND context is preempted by RTC_0_ to start the FENP task *μ_j_* and then *μ_k_*. At *t*_8_, the BGND context is restored and then interrupted again at *t*_9_ for the MEDF task *ε_r_*.

It is worth mentioning that this layered approach offers isolation between the criticality levels in the sense that the highest *(*“*extreme*”*)* criticality level is isolated from all the other levels, while the “*high*” criticality level offers isolation between tasks with the same criticality level and from the “*low*” criticality level tasks.

### 4.4. Runtime Overhead Analysis

Unlike many approaches in the literature, system overhead is not ignored, nor considered negligible in our work. To accommodate runtime task scheduling and execution, specific mechanisms must be implemented for each of the three existing contexts. From the practical experience, including the development of the HARETICK kernel and the corresponding real-time application set, these mechanisms account for a relatively significant processing effort.

(a) FENP execution context is supported by the online scheduler and the task dispatcher. Runtime scheduling of the FENP tasks is carried out, based on periodic scheduling cycles (or frames), by a scheduling task, which fills a Dispatch Table with the task IDs and start times of each scheduling cycle. The runtime scheduler is also a FENP task, part of the ***S****^FENP^* subset (*μ_j_* in Figure 1, for example), and thus it takes itself too into consideration when applying the scheduling algorithm.

The worst-case execution time of the FENP task dispatcher, briefly presented in the previous subsection, is not negligible either. Therefore it is added to the computational cost (*C_i_*) of each task *μ_i_* (*i* = 1, …, *m*) at the offline schedulability analysis phase. A detailed discussion of the FENP runtime mechanisms, including implementation-specific values and experimental measurements, is provided in [2,27].

(b) MEDF execution context is supported by the online scheduling module, which is called upon the completion of each MEDF job. It serves also as a dispatcher, as it prepares the execution of the next job, either by launching it immediately, if the job is ready, or by programming the RTC_1_ timer interrupt, as in the case of ε_r_ in Figure 1. The execution time of the MEDF scheduling module is added to the computational cost of each task ε*_j_* (*j* = 1…*n*) during offline system schedulability analysis and is also considered when scheduling each MEDF task at runtime.

This approach puts significant pressure on the efficient programming and optimization techniques necessary to reduce as much as possible the worst-case execution time of the runtime scheduling and execution support mechanisms previously described.

## 5. Integrated Schedulability Analysis of H^2^RTS

### 5.1. Preliminaries

Schedulability analysis of real-time systems has been developed in two distinct directions: exact tests and polynomial sufficiency test [35,36]. The advantages of an exact test are obvious, as the test can be applied to any set of tasks, to check if the set is schedulable or not. Unfortunately, these kinds of tests are extremely complex (usually of non-polynomial complexity) and can only be used for offline analysis.

On the other hand, sufficiency tests usually have a smaller complexity, but they offer only the sufficient conditions, meaning that there can be found sets of tasks which fail the test, but can be scheduled with the given algorithm.

Considering the system ***S*** of hard real-time tasks described by Equation (1), an essential question is how to determine if its operation is feasible according to the H^2^RTS method proposed here. As the FENP tasks in the ***S**^FENP^* subset run in the highest priority execution context, they are schedulable according to the tests described in [2], without any potential influence other tasks in ***S*** might have on the system timing behavior. Thus, the H^2^RTS schedulability problem reduces to finding a feasible schedule to the *ε_j_* (*j* = 1…*n*) tasks in the ***S****^MEDF^* subset over a time interval of arbitrary length, while also considering the corresponding workload required by the FENP tasks over the same interval.

Due to the particularities of the MEDF algorithm, its schedulability analysis must take into account the inherent contradiction regarding the task execution within this context. On the one hand, an *ε_j_* (*j* = 1…*n*) task cannot be preempted by any other *ε_k_* (*k* ≠ *j*) task, therefore, MEDF behaves like a non-preemptive EDF. On the other hand, a MEDF task *ε_j_* can be preempted by any task *μ_i_* (*i* = 1…*m*) from the FENP context. From this perspective, the MEDF algorithm resembles to the classical, preemptive EDF scheduling technique. As a result, the schedulability analysis techniques currently established in the field for both the preemptive and the non-preemptive EDF algorithms must be reconsidered and adapted to the particularities of the MEDF method.

**Figure 2:**
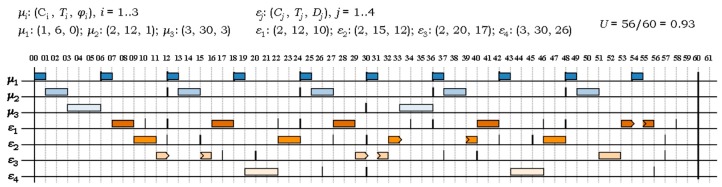
H^2^RTS scheduling of a system of *N* = 7 hard real-time tasks.

Figure 2 illustrates the H^2^RTS scheduling method for a system of *m* = 3 FENP tasks, ***S****^FENP^* = {*μ*_1_, *μ*_2_, *μ*_3_}, and *n* = 4 MEDF tasks, ***S****^MEDF^* = {*ε*_1_, *ε*_2_, *ε*_3_, *ε*_4_}. Their respective timing parameters, as presented in Section 4.2 and Section 4.3, are specified in the figure. Task periods are depicted with thicker lines, while the deadlines are represented with thinner lines. The total utilization factor is *U* = 0.93 and, as shown in Figure 2, this particular system is schedulable under the H^2^RTS algorithm.

### 5.2. Schedulability Analysis of the H^2^RTS Algorithm

In this section, we will derive the tests and conditions which establish if a MEDF task, *ε_j_* (*j* = 1...*n*), will meet its deadline under the worst-case operation of the considered system, ***S*** ≡ {***S****^FENP^*, ***S****^MEDF^*}.

**Definition** **1.** *The execution intervals of a task τ_i_, cumulated over a time interval [0, t] under the worst-case scenario, is defined as the worst-case workload of task τ_i_ and denoted with w_i_(t). Further, with W_i_(t) we denote the worst case workload of the top i tasks, which is the cumulated workload of the i highest priority tasks over the time interval [0, t]:*

(8) $$W_{i}\left(t\right)=\sum_{j=1}^{i}w_{j}\left(t\right)$$

**Definition** **2.** *The worst-case processor demand of a task τ_i_, denoted as h_i_(t), is the maximum amount of processing time which can be required under the worst-case scenario by τ_i_ over a time interval [0, t]*.

Due to the non-preemptive execution paradigm employed by both scheduling algorithms that compose the H^2^RTS mechanism, the worst-case processor demand of any task in ***S*** can be expressed as

(9) $$h_{i}\left(t\right)=\left\lceil \frac{t}{T_{i}}\right\rceil C_{i}\;,\;\forall \mu_{i}\in S^{FENP}\;\wedge \forall \varepsilon_{i}\in S^{MEDF}$$

**Theorem** **1.** *A task set **S** = {**S**^FENP^, **S**^MEDF^}, with* $S^{FENP}=\left\{\;\mu_{i}\equiv \left(\phi_{i}^{\mu },\;C_{i}^{\mu },\;T_{i}^{\mu }\right)\;|\;i=1..\;m\right\}$ *and* $S^{MEDF}=\left\{\;\varepsilon_{j}\equiv \left(C_{j}^{\varepsilon },\;T_{j}^{\varepsilon },\;D_{j}^{\varepsilon }\right)\;|\;j=1..\;n\right\}$ *, is schedulable with the H^2^RTS algorithm if:*

*(a)*  **S**^FENP^ is feasible, and
*(b)*  (10) $$C_{j}^{\varepsilon }+\sum_{i=1}^{m}\left\lceil \frac{D_{j}^{\varepsilon }}{T_{i}^{\mu }}\right\rceil \;C_{i}^{\mu }+\sum_{p=1}^{j-1}\left\lceil \frac{D_{j}^{\varepsilon }}{T_{p}^{\varepsilon }}\right\rceil \;C_{p}^{\varepsilon }+\underset{j<r\leq \;n}{\max}C_{r}^{\varepsilon }\;\leq D_{j}^{\varepsilon }\;,\;\forall \;j=1...n$$

**Proof.**  Condition (a) is treated in details by the tests described in [2]. The second point states that if any task *ε_j_* ∈ ***S**^MEDF^* satisfies Condition (10), it can be scheduled with the H^2^RTS algorithm. Considering an arbitrary job of the *ε_j_* task, *ε_j_*_,*k*_, its execution is feasible if it does not exceed the corresponding deadline, $d_{j,k}=kD_{j}^{\varepsilon }$. In terms of worst-case workloads, this is equivalent to

(11) $$w_{j}\left(t\right)+W^{FENP}\left(t\right)+W_{\mathrm{hp}}^{MEDF}\left(t\right)+W_{\mathrm{lp}}^{MEDF}\left(t\right)\leq t\;.\;t=d_{j,k}$$

where, *w_j_*(*t*) is the workload of *ε_j_*, *W^FENP^*(*t*) is the workload of all the tasks in the ***S****^FENP^* subset, since they have precedence over the *ε_j_* task, $W_{\mathrm{hp}}^{MEDF}\left(t\right)$ is the cumulated workload of all the MEDF tasks with higher priority than *ε_j_*, and $W_{\mathrm{lp}}^{MEDF}\left(t\right)$ is the workload corresponding to the lower-priority MEDF tasks which can resume execution after a context switch, from FENP to MEDF, in a time interval which can be critical for *ε_j_*. □

**Figure 3:**
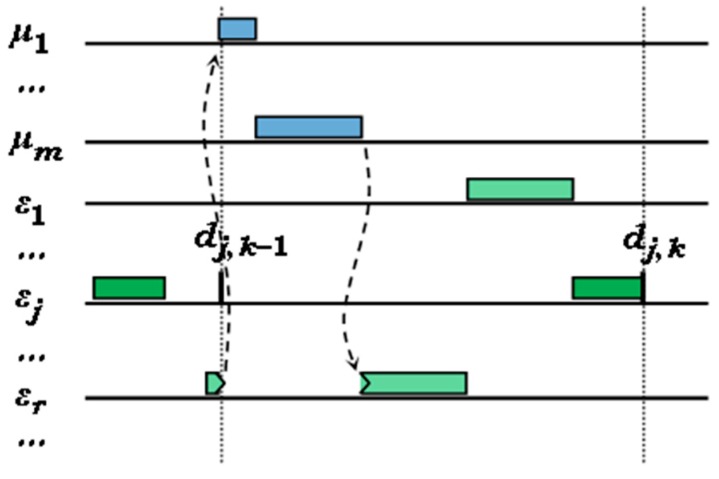
Higher priority MEDF task blocked by a lower priority one, previously interrupted by the FENP context.

The $W_{\mathrm{lp}}^{MEDF}\left(t\right)$ scenario derives from the MEDF scheduling particularity presented in Section 4.3, which allows the higher precedence FENP context to preempt the execution of any MEDF task, but, when the MEDF context is restored back, the same task will resume execution regardless of the fact another MEDF task, with higher priority, could become ready in the meanwhile. Figure 3 exemplifies this situation, which can be considered somewhat similar to a blocking time for the *ε_j_* task in priority inversion phenomena, typically found in preemptive systems with concurrent resource accesses.

Taking into account that the time interval for calculating the workloads is $t=kD_{j}^{\varepsilon }$, Equation (11) is further equivalent to

(12) $$kC_{j}^{\varepsilon }+W^{FENP}\left(kD_{j}^{\varepsilon }\right)+W_{\mathrm{hp}}^{MEDF}\left(kD_{j}^{\varepsilon }\right)+k\underset{j<r\leq \;n}{\max}C_{r}^{\varepsilon }\leq kD_{j}^{\varepsilon }$$

where, for the lower-priority MEDF tasks workload, $W_{\mathrm{lp}}^{MEDF}\left(t\right)$, the task with the longest worst-case execution time has been considered. On the other hand, the real workload of a set of tasks over a time interval is bounded from above by their corresponding worst-case processor demand: *w_i_*(*t*) ≤ *h_i_*(*t*). Using Equations (8) and (9), Relation (12) becomes:

(13) $$kC_{j}^{\varepsilon }+\sum_{i=1}^{m}\left\lceil \frac{kD_{j}^{\varepsilon }}{T_{i}^{\mu }}\right\rceil \;C_{i}^{\mu }+\sum_{p=1}^{j-1}\left\lceil \frac{kD_{j}^{\varepsilon }}{T_{p}^{\varepsilon }}\right\rceil \;C_{p}^{\varepsilon }+k\underset{j<r\leq \;n}{\max}C_{r}^{\varepsilon }\leq kD_{j}^{\varepsilon }$$

Based on the known property of the ceiling function, stating that ⌈x+y⌉≤⌈x⌉+⌈y⌉, the second term in Equation (13) can be expressed as

(14) $$\sum_{i=1}^{m}\left\lceil \frac{kD_{j}^{\varepsilon }}{T_{i}^{\mu }}\right\rceil \;C_{i}^{\mu }\leq k\sum_{i=1}^{m}\left\lceil \frac{D_{j}^{\varepsilon }}{T_{i}^{\mu }}\right\rceil \;C_{i}^{\mu }$$

and the third term can be bounded in a similar way. As a result, Equation (13) becomes equivalent to Equation (10), which is the relation to be proved.

Theorem 1 provides a sufficiency condition for the schedulability test of MEDF tasks. It basically states that if the workload for any MEDF task over a time interval equal to its relative deadline is less than or equal to this deadline, the ***S***^MEDF^ task subset can be feasibly scheduled and executed within the H^2^RTS system. Theorem 1 approximates the workload with an upper bound function, i.e., the worst-case processor demand. From the algorithmic implementation perspective, this upper bound function is non-linear and involves the computation of the ceiling operator, which complicates the schedulability test of each MEDF task in the system.

**Theorem** **2.** *A task set **S** = {**S**^FENP^, **S**^MEDF^}, with* $S^{FENP}=\left\{\;\mu_{i}\equiv \left(\phi_{i}^{\mu },\;C_{i}^{\mu },\;T_{i}^{\mu }\right)\;|\;i=1..\;m\right\}$ *and* $S^{MEDF}=\left\{\;\varepsilon_{j}\equiv \left(C_{j}^{\varepsilon },\;T_{j}^{\varepsilon },\;D_{j}^{\varepsilon }\right)\;|\;j=1..\;n\right\}$, *is schedulable with the H^2^RTS algorithm if:*

*(a)*  **S**^FENP^ is feasible, and
*(b)*  (15) $$\frac{C_{j}^{\varepsilon }+\sum_{i=1}^{m}C_{i}^{\mu }\left(1-U_{i}^{\mu }\right)+\sum_{p=1}^{j-1}C_{p}^{\varepsilon }\left(1-U_{p}^{\varepsilon }\right)+\underset{j<r\leq \;n}{\max}C_{r}^{\varepsilon }}{1-\sum_{i=1}^{m}U_{i}^{\mu }-\sum_{p=1}^{j-1}U_{p}^{\varepsilon }}\leq D_{j}^{\varepsilon }\;,\;\forall \;j=1..\;n$$

**Proof.**  Following the same steps as in Theorem 1, Relation (12) is reached. Further, the workload corresponding to all the tasks in the ***S**^FENP^* subset, *W^FENP^*(*t*), can be bounded from above with the linear function proposed in [37]:

(16) $$W^{FENP}\left(t\right)\leq \sum_{i=1}^{m}\left(U_{i}^{\mu }t+C_{i}^{\mu }\left(1-U_{i}^{\mu }\right)\right)$$

As $t=kD_{j}^{\varepsilon }$ (*k* = 1, 2, ...), (16) is equivalent to

(17) $$W^{FENP}\left(kD_{j}^{\varepsilon }\right)\leq \sum_{i=1}^{m}\left(U_{i}^{\mu }kD_{j}^{\varepsilon }+C_{i}^{\mu }\left(1-U_{i}^{\mu }\right)\right)\leq k\sum_{i=1}^{m}\left(U_{i}^{\mu }D_{j}^{\varepsilon }+C_{i}^{\mu }\left(1-U_{i}^{\mu }\right)\right)$$

A similar bound can be applied to the cumulated workload of all the MEDF tasks with higher priority than *ε_j_*, $W_{\mathrm{hp}}^{MEDF}\left(t\right)$:

(18) $$W_{\mathrm{hp}}^{MEDF}\left(kD_{j}^{\varepsilon }\right)\leq \sum_{p=1}^{j-1}\left(U_{p}^{\varepsilon }kD_{j}^{\varepsilon }+C_{p}^{\varepsilon }\left(1-U_{p}^{\varepsilon }\right)\right)\leq k\sum_{p=1}^{j-1}\left(U_{p}^{\varepsilon }D_{j}^{\varepsilon }+C_{p}^{\varepsilon }\left(1-U_{p}^{\varepsilon }\right)\right)$$

replacing the terms from Equations (17) and (18) into Equation (12) gives

(19) $$C_{j}^{\varepsilon }+\sum_{i=1}^{m}\left(U_{i}^{\mu }D_{j}^{\varepsilon }+C_{i}^{\mu }\left(1-U_{i}^{\mu }\right)\right)+\sum_{p=1}^{j-1}\left(U_{p}^{\varepsilon }D_{j}^{\varepsilon }+C_{p}^{\varepsilon }\left(1-U_{p}^{\varepsilon }\right)\right)+\underset{j<r\leq \;n}{\max}C_{r}^{\varepsilon }\leq D_{j}^{\varepsilon }\;,\;\forall \;j=1..\;n$$

which is equivalent to Relation (15), the statement of the theorem. □

**Figure 4:**
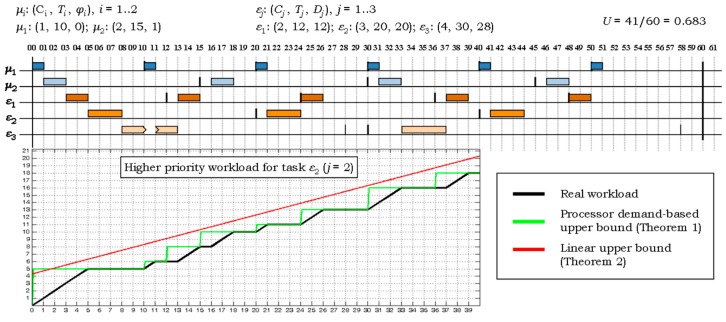
Example system of *N* = 5 hard real-time tasks: its H^2^RTS schedule and the higher priority workload for the task *ε*_2_.

Figure 4 presents the H^2^RTS scheduling of a hard real-time system composed of 2 FENP and 3 MEDF tasks. It also illustrates in a comparative manner the real workload and its upper bound functions, calculated according to Theorems 1 and 2, for the tasks of higher priority than the MEDF task *ε*_2_. As the figure also shows, the upper bound of the workload based on the processor demand (Theorem 1) is closer to the real workload, i.e., it better approximates the real workload as the upper bound function stated in Theorem 2. As a result, the former sufficiency test will reject fewer task systems which are in fact feasible under the H^2^RTS algorithm. On the other hand, Relation (15) is linear and avoids the ceiling operations required by the test in Theorem 1. Furthermore, it makes use of intermediate iterative sums of the form ∑*_i_ C_i_*(1 − *U_i_*) and ∑*_i_ U_i_*, which can be computed at each step *i* by accumulating the current term to the result of the previous step, thus avoiding the recalculation of the entire sum at each iteration.

The sufficiency tests stated by the two theorems can be rewritten in a more compact form by using a uniform notation for the entire task system in Equation (1):

(20) $$\begin{array}{l} S=\left\{S^{FENP},\;S^{MEDF}\right\}=\left\{\tau_{i}|\;i=1..N\right\}\;,\\ \{\begin{array}{l} \tau_{i}\in S^{FENP},\;\tau_{i}\equiv \left(\phi_{i},\;C_{i},\;T_{i}\right),\;1\leq i\leq m\\ \tau_{i}\in S^{MEDF},\;\tau_{i}\equiv \left(C_{i},\;T_{i},\;D_{i}\right),\;m<i\leq N \end{array} \end{array}$$

Then, the sufficiency test Equation (10) for MEDF tasks in Theorem 1 becomes

(21) $$C_{j}+\sum_{i=1}^{j-1}\left\lceil \frac{D_{j}}{T_{i}}\right\rceil \;C_{i}+\underset{j<r\leq \;N}{\max}C_{r}\;\leq D_{j}\;,\;\forall \;j.\;m<j\leq N$$

and the sufficiency test Equation (15) in Theorem 2 is equivalent to

(22) $$\frac{C_{j}+\sum_{i=1}^{j-1}C_{i}\left(1-U_{i}\right)+\underset{j<r\leq \;n}{\max}C_{r}}{1-\sum_{i=1}^{j-1}U_{i}}\leq D_{j}\;,\;\forall \;j\;.\;m<j\leq N$$

We note that the feasibility test in Theorem 2, based on the linear upper bound of the worst case workload, as stated by Equation (22), is consistent with the results of Bini, Nguyen, Richard and Baruah for the case of fixed priority sporadic task systems scheduled with preemptive mechanisms and including blocking time penalties [37].

## 6. Performance Evaluation and Experimental Results

### 6.1. Performance Analysis

To analyze the performance of the H^2^RTS, a set of specialized software programs have been implemented.

“Parameter Task Generator” is used for generating test sets of hard real-time tasks. The program allows the user to specify the interval limits for the task periods and the total processor utilization factor for each task set. Thus, the period *T_i_* and computational cost *C_i_* of each task *τ_i_* will be randomly generated to fulfill the specified conditions. A uniform distribution has been chosen for the generation of the *T_i_* parameter and, additionally, an option to choose a greatest common divider (GCD) for the periods has been provided, to establish a harmonic relationship between them and, thus, to increase the occurrence probability of feasible task systems. As the generated task parameters can significantly influence the results and conclusions drawn when analyzing schedulability tests, the configuration options of the Parameter Task Generator take into consideration the following aspects of a proper simulation [35]:

- The task period, *T_i_*, is usually defined by the user; therefore, treating periods as random numbers does not reflect the real situations. Moreover, tasks can have harmonic relations between their periods which cannot be reflected by random numbers.
- The computation time, *C_i_*, depends on the platform where the application runs and it cannot be estimated without a prior knowledge of the tasks. Moreover, to consider a uniform distribution of the *C_i_* on the [0, *T_i_*] interval is equivalent to consider the processor utilization factor *U_i_* uniform on the [0, 1] interval.
- The processor utilization factor of a task, *U_i_*, is proposed to be taken into consideration, because of the numerous algorithms that actually depend on it. In addition, a randomly generated uniform distribution of *U_i_* is closer to the real situations.

“H^2^RTS Simulator” has been designed to receive as input a set of tasks to be simulated, specified by its time parameters. The output is a log file with the simulation results and a test analysis for the respective system of tasks. The simulator features two types of log files: a detailed log file containing the launch time instance and the index of each scheduled task, along with the preemption and task resume events, and a brief log file with the simulation results and test analysis in terms of PASS/FAIL.

A total of over 22,000 hard real-time task sets have been simulated, covering the intervals of interest for their main parameters, as shown in Table 1. The value specified as greatest common divisor (GCD) of the FENP task periods simulates the harmonic relationship that usually exists among the periods of such tasks, as mentioned above. The additional conditions stated in Equation (23) eliminate the randomly generated tasks which are not feasible from the start, i.e., tasks with execution times larger than the shortest task periods in the same category (subset).

**Figure 5:**
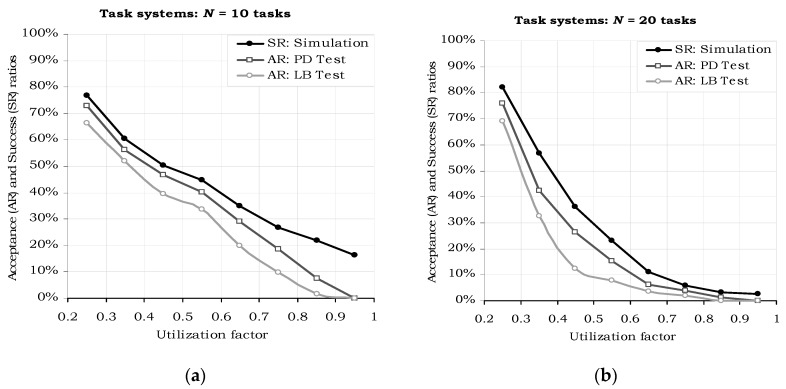
Acceptance ratios (AR) and success ratios (SR) versus total utilization factor. (**a**) *N* = 10 tasks, (**b**) *N* = 20 tasks.

Figure 5 presents the results of the acceptance tests and of the actual execution simulations for sets of *N* = 10 (a) and *N* = 20 tasks (b), at various total utilization factors. “PD Test” denotes the “Processor Demand” acceptance test, which is specified by the sufficiency condition Equation (10) introduced by Theorem 1. “LB Test” is the “Linear Bound” acceptance test, specified by Theorem 2 with the sufficiency condition Equation (15). All the simulations revealed the correct behavior of the proposed sufficiency tests, i.e., all the generated task sets that passed the tests have been successfully scheduled and executed with the H^2^RTS algorithm. The acceptance test plot lines (the “AR” lines) are positioned below the simulation results (the “SR” lines) in all the evaluations performed.

**Figure 6:**
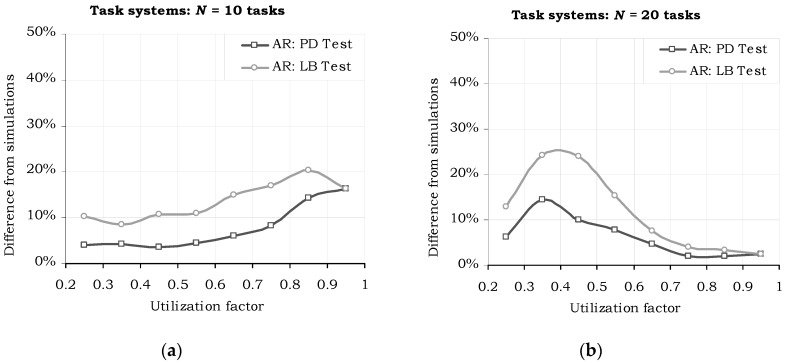
Difference between the simulation results and the acceptance test results, at various total utilization factors. (**a**) *N* = 10 tasks, (**b**) *N* = 20 tasks.

Another important observation drawn from the graphs in Figure 5 is related to the higher effectiveness of the PD acceptance test as compared to the “Linear Bound” test: the graph line of the former is closer to the real (simulated) success ratio, than the latter, meaning it accepts more task sets which are eventually schedulable with the H^2^RTS algorithm. Figure 6 emphasizes this particular performance aspect, by depicting the difference between the simulated and the acceptance test results. This fact is directly derived from the discussion below the proof of Theorem 2 and the example in Figure 4. The tradeoff regarding the acceptance tests is done between the accuracy and the complexity of these tests. Processor Demand acceptance test is more accurate, but requires a more complex computation, which results in a higher overhead, while Linear Bound test is simpler but less accurate. Thus, PD Test is suitable for offline computation, while LB Test can be used in online systems, even if the systems have restricted computation capabilities.

**Figure 7:**
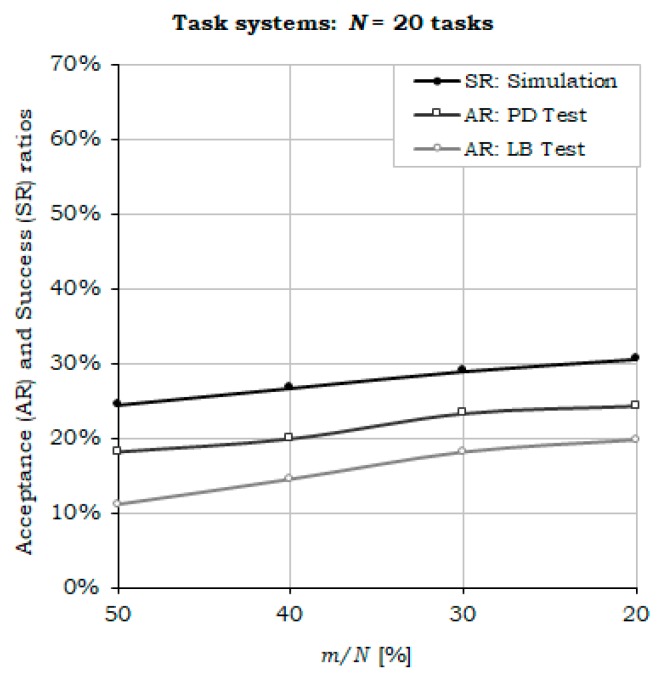
Acceptance and success ratios, while decreasing the proportion of FENP tasks (*m*/*N* ratio).

**Figure 8:**
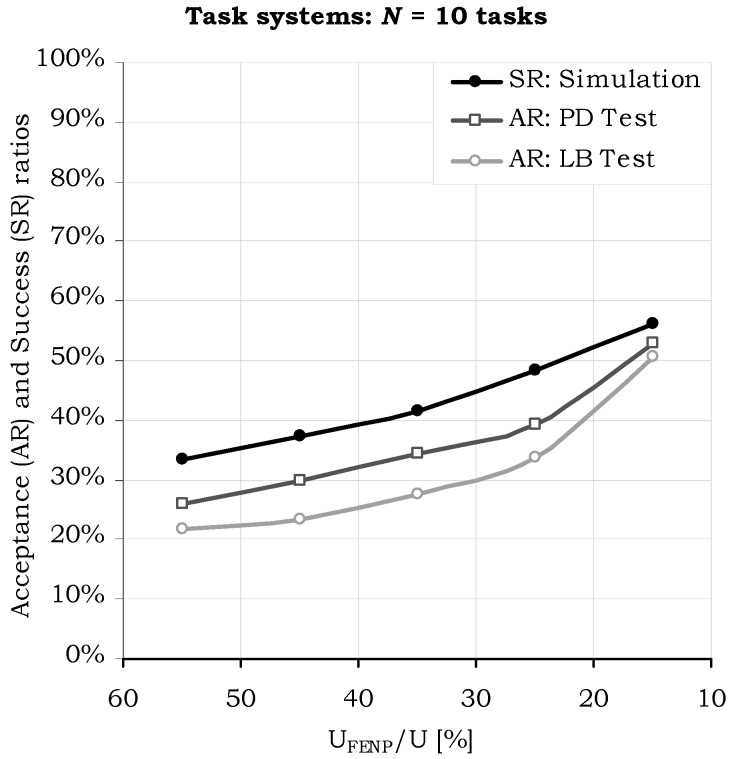
Sufficiency tests and simulation results, while decreasing the utilization factor of the FENP tasks (*U_FENP_*/*U* ratio).

**Figure 9:**
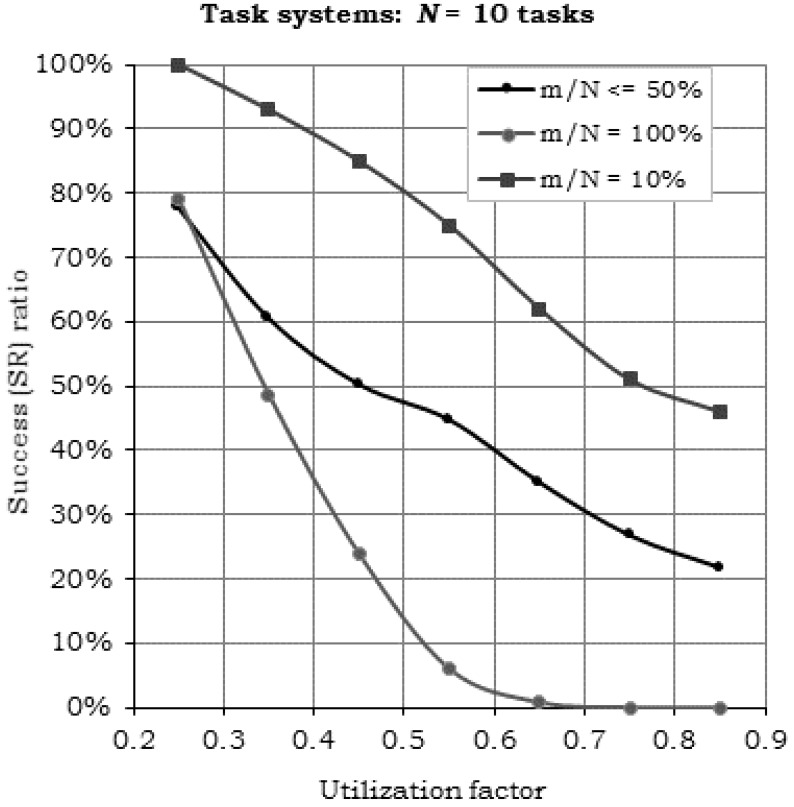
Simulation results when varying the *m*/*N* ratio.

The results depicted in Figure 7, Figure 8 and Figure 9 confirm the whole reasoning of designing the H^2^RTS as a hybrid scheduling technique, by introducing a second execution context, the MEDF algorithm, to increase the performance of the FENP context in terms of processor utilization, while preserving its high predictability features. The graphs show that, by decreasing the weight of FENP tasks within the task set ***S*** (the ratio *m*/*N*, where *m* represents the number of FENP tasks and *N* represents the total number of tasks in the set ***S***) (Figure 7), or processor utilization factor (Figure 8), the success ratio increases in a proportional manner. 

Simulation results for the variation of the *m/N* ratio for sets of 10 hard real-time tasks, presented in Figure 9 show:

- Compared to the case, where *m*/*N* = 10%, H^2^RTS method (*m*/*N* ≤ 50%) provides up to 50% jitter-less task execution, but reduced the success ratio by a factor of 24.25%.
- Compared with the limit case, when ***S*** = ***S****^FENP^* (*m*/*N* = 100%), H^2^RTS offers an increase of success ratio with 21.79%, but a decrease in the number of tasks with jitter-less execution up to 50%.

### 6.2. Experimental Validation

The H^2^RTS scheduling technique has been successfully implemented and tested on a set of sensor networks and robotic systems with hard real-time operating specifications developed at the DSPLabs Timisoara, especially within the experimental CORE-TX platform [38].

From the practical experience, one of the best validation environments for hard real-time systems and scheduling mechanisms are applications which communicate over synchronous interfaces, such as the SPI (Serial Peripheral Interface). The SPI is a commonly used interface to send data between the microcontroller and different sensors like temperature sensors, barometric pressure sensors, video cameras and others.

**Figure 10:**
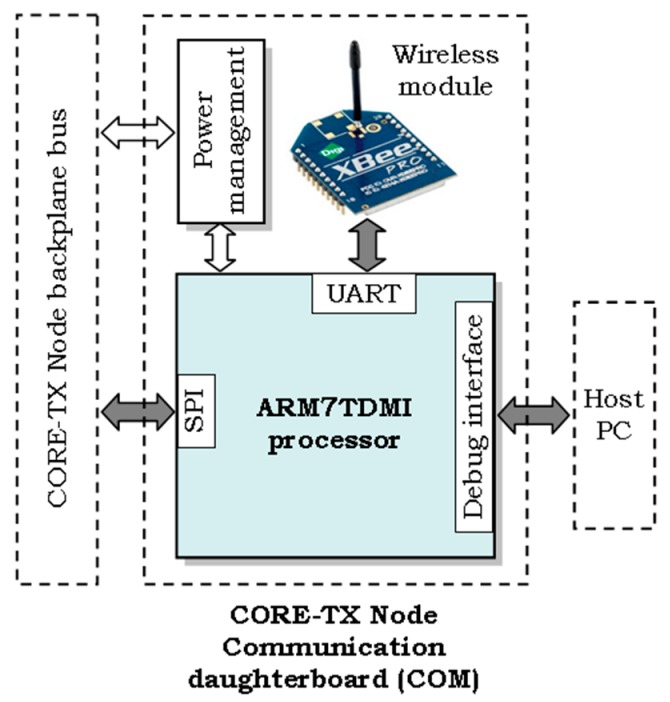
Block diagram of the CORE-TX node Communication board.

Our experimental setup is exemplified in Figure 10, as the wireless communication daughter board of a CORE-TX node, which is based on an upgraded version of the XBee system presented in [29]. The central processing and control unit of the communication board, implemented with an ARM7TDMI processor, operates under the HARETICK kernel, based on the H^2^RTS hybrid scheduling mechanism. A set of important tasks are programmed on the onboard processor, including:

- Communication with the other CORE-TX node boards, including the motherboard, which generates and processes the wireless data at node level. The SPI synchronous interface is used by this task and, therefore, it must be scheduled with hard real-time constraints to obtain a minimum execution jitter.
- Control of and communication with the onboard wireless module (an XBee module in our case), to provide the necessary data exchanges with the other nodes of the wireless network. This task uses the UART interface, which also needs to be operated in a hard real-time manner. Nevertheless, due to its asynchronous behavior and relatively low bit rates, it does not require a perfectly synchronous operation as in the case of the SPI task.
- Exchange and process debug and execution trace information with a host PC, to provide additional experimental data, besides the direct measurements performed with the oscilloscope and the logic analyzer. To prevent loss of debug and trace information during the exchanges with the host PC, this task is executed with synchronous, hard real-time specifications, similarly to the SPI task.
- Various, local data processing and control operations are included in a common processing task, which does not require real-time execution.

The most relevant system and task configuration parameters of this experimental setup are synthesized in Table 2 below.

The correct execution of the system has been validated through a large number of various echo-type data communication tests and its exact behavior in time has been verified and confirmed by extensive measurements using the logic analyzer.

**Figure 11:**
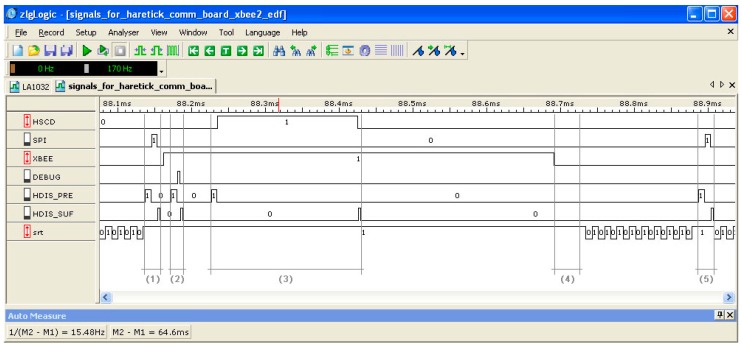
Operation of the XBee-based wireless board, captured with the logic analyzer.

An example timeframe caption of the H^2^RTS-based system operation is presented in Figure 11. Here, the execution of the SPI and the DEBUG FENP tasks is shown, each of them being framed by the Dispatch prefix (HDIS_Pre) and suffix (HDIS_Suf) components of the FENP execution context (see intervals denoted with (1) and (2), respectively, in Figure 11). Interval (3) highlights an execution instance of the FENP Scheduler (HSCD), also framed by the Dispatch components, as it is considered and scheduled as a FENP task itself by the system. With (4), the execution of the MEDF scheduler is captured, following the termination of an instance of the XBee MEDF task. This logic analyzer caption also shows the behavior of the system during the execution of a MEDF task: the XBee task starts at some scheduled instance after the execution of the SPI FENP task (1) and it is interrupted by other FENP tasks, such as the DEBUG (2) and the HSCD (3), due to their precedence over the MEDF context. Finally, the background, SRT task is shown at the bottom of the timeframe caption, with the lowest execution priority.

Comparing the proposed hybrid scheduling architecture with the FENP scheduling approach described in [29], we notice a significant improvement in total processor utilization factor for real-time tasks from 37.1% to 69.59% (for the above-mentioned scenario). In the same circumstances, the overall scheduling overhead will be degraded, but remains at a satisfactory level (under 1 ms).

## 7. Conclusions

This paper proposes a new hybrid scheduling algorithm, called H^2^RTS, which is particularly suitable for sensor nodes that run periodic or sporadic hard, firm or soft real-time tasks, defined both in a strict (i.e., with a perfectly periodical, jitter-less execution) or in a more general sense (i.e., the time between the executions of two successive jobs of the same task is greater or equal to the task period and smaller than the double of the same task period).

The key feature of the H^2^RTS technique is its hybrid design, which combines an offline static scheduling algorithm with a dynamic one, thus gaining the advantages of predictability and determinism from the static algorithm, and the flexibility and increased acceptance ratio from the dynamic technique. As resulting from the analysis of H^2^RTS, it is able to schedule and execute tasks in a deterministic jitter-less manner, as the standalone FENP mechanism, while also providing a much higher overall acceptance ratio for real-time applications. Furthermore, a set of sufficiency tests have been introduced and demonstrated for the proposed scheduling technique, based on the processor demand and on a linear upper bound.

The performance and correct behavior of the proposed H^2^RTS hybrid scheduling technique and its corresponding sufficiency tests have been extensively evaluated and validated both on a simulator and on a sensor mote equipped with ARM7 microcontroller. The evaluation results show that, by employing this hybrid scheduling along with an a priori partitioning of the application set of tasks, the system is able to increase its schedulability success ratio in average by 21.79%, for sets of 10 tasks, and 11.33% for sets of 20 tasks, while maintaining the same predictability and jitter-less execution of critical tasks, as compared to a static, table-driven scheduling scheme.

As future work, we plan to extend this algorithm by including the possibility of dynamically task migration from one criticality level to another and to deepen the schedulability analysis for this new extension.

## Figures and Tables

**Table 1:** Main setup parameters of the simulations.

Parameter	Value/Range
Total utilization factor	*U* = 0.2/0.95
Size of task sets (FENP + MEDF tasks)	*N* = *m* + *n* = {5, 10, 20}
FENP tasks ratio	*m*/*N* = {20%, 30%, 40%, 50%}
Processor utilization factor of FENP tasks versus total utilization factor	*UFENP/U* = 10%/60%
Interval for generating the task periods	*T_i_* = 10/510
Algorithm for generating the task periods	Random, with uniform distribution
Greatest common divisor of FENP task periods	GCD = 30
Algorithm for generating the task execution times	Random, with uniform distribution
Additional conditions for generating the task execution times	$\{\begin{array}{c} \underset{1\;\leq \;j\;\leq \;m}{\max}C_{j}^{\mu }\leq \underset{1\;\leq \;i\;\leq \;N}{\min}T_{i}\\ \underset{1\;\leq \;j\;\leq \;n}{\max}C_{j}^{\varepsilon }\leq \underset{1\;\leq \;i\;\leq \;n}{\min}T_{i}^{\varepsilon } \end{array}$(23)

**Table 2:** Main system and task configuration parameters of the CORE-TX based experimental setup.

Parameter	Value/Range
ARM7TDMI CPU clock	58.9824 MHz
HARETICK Real-Time Clock (RTC)	14.7456 MHz
XBee UART transfer rate	57,600 bps
FENP execution environment:	
Worst case execution time (WCET) of the FENP Dispatch Prefix (HDIS_Pre)	112 RTC cycles (7.59 μs)
WCET of the FENP Dispatch Suffix (HDIS_Suf)	68 RTC cycles (4.61 μs)
WCET of the FENP Scheduler (HSCD)	4052 RTC cycles (274.79 μs)
SPI communication task WCET (FENP task)	310 RTC cycles (21 μs)
DEBUG task WCET (FENP task)	605 RTC cycles (41 μs)
XBee task minimum frequency (MEDF task)	360 Hz (considering a UART FIFO buffer of 16 frames and 10 bits per frame)
XBee task WCET	4568 RTC cycles (309.79 μs)
Common low priority processing task, including LED toggle (SRT/BGND task)	No timing specifications
